# Soluble Polysaccharide Derived from *Laminaria japonica* Attenuates Obesity-Related Nonalcoholic Fatty Liver Disease Associated with Gut Microbiota Regulation

**DOI:** 10.3390/md19120699

**Published:** 2021-12-09

**Authors:** Yiping Zhang, Longhe Yang, Nannan Zhao, Zhuan Hong, Bing Cai, Qingqing Le, Ting Yang, Lijun Shi, Jianlin He

**Affiliations:** 1 Third Institute of Oceanography, Ministry of Natural Resources, Xiamen 361005, China; ypzhang@tio.org.cn (Y.Z.); longheyang@tio.org.cn (L.Y.); tiffiny1104@163.com (N.Z.); hzh@tio.org.cn (Z.H.); caibing@tio.org.cn (B.C.); leqingqing@tio.org.cn (Q.L.); 18965156193@189.cn (T.Y.); slj15711567715@163.com (L.S.); 2 Technical Innovation Center for Exploitation of Marine Biological Resources, Ministry of Natural Resources, Xiamen 361005, China

**Keywords:** obesity, brown seaweed, polysaccharide, nonalcoholic fatty liver disease, gut microbiota

## Abstract

In this study, the effects of a polysaccharide derived from *Laminaria japonica* (LJP) on obesity were investigated in mice fed a high-fat diet (HFD). LJP significantly attenuated obesity-related features, lowering serum triglycerides, glucose, total cholesterol and low-density lipoprotein cholesterol levels. HFD-induced liver steatosis and hepatocellular ballooning were significantly attenuated by LJP. Additionally, LJP was found to significantly modulate hepatic gene expressions of AMPK and HMGCR, which are key regulators of lipid and cholesterol metabolism. We further found that LJP ameliorated HFD-induced gut microbiota (GM) dysbiosis by significantly reducing the obesity-related Firmicutes to Bacteroidetes ratio, meanwhile promoting the growth of Verrucomicrobia at the phylum level. At the genus level, propionate-producing bacteria *Bacteroides* and *Akkermansia* were elevated by LJP, which might explain the result that LJP elevated fecal propionate concentration. Taken together, these findings suggest that dietary intake of LJP modulates hepatic energy homeostasis to alleviate obesity-related nonalcoholic fatty liver disease associated with GM regulation.

## 1. Introduction

Worldwide obesity has nearly tripled since 1975 [1]. The prevalence of obesity has raised a global concern over the challenge to prevent chronic diseases, such as heart disease, diabetes, high blood pressure and certain cancers [2]. Notably, nonalcoholic fatty liver disease (NAFLD) is rapidly becoming the most common cause of chronic liver disease due to an increase in the prevalence of obesity [3].

The pathophysiology of NAFLD is a complex process that involves dietary factors, insulin resistance, genetic polymorphisms and lipotoxicity. Recent studies have implicated the gut microbiota (GM) as a critical player, since it modulates nutrient uptake, energy homeostasis and chronic metabolic disorders [4]. Considerable evidence that GM dysbiosis contributes to the pathogenesis of NAFLD has been provided by animal and human studies. A potential causal role of dysbiosis on NAFLD has been suggested. Dysbiosis increases gut permeability and may increase hepatic exposure to injurious substances that increase hepatic inflammation and fibrosis [5]. An unhealthy diet is one of the key risk factors of NAFLD. On the one hand, GM actively participates in food digestion and facilitates the absorption of dietary molecules. On the other hand, dietary components provide nutrients for bacteria, which then produce metabolites involved in energy balance, metabolism and immune response. The interplay between poor diet and dysbiosis changes the metabolism of food substrates, impacting the pathophysiology of NAFLD [6]. Diet quality can be improved by reducing the consumption of energy-dense foods and by increasing the intake of dietary fiber, since the composition and diversity of the GM can be improved by dietary fiber intake [7]. The brown seaweed *Laminaria japonica* (*Saccharina japonica*) is a traditional cuisine in Japan, Korea and China, favored on account of its unique flavor and high nutritional value. It contains minerals, such as magnesium, iodine, calcium, iron and zinc, as well as fat-soluble components, e.g., fucoxanthin and fucosterol, and is particularly rich in soluble fibers, e.g., alginate and fucoidan [8]. In recent years, *L. japonica* has attracted attention in relation to the prevention and treatment of lifestyle-related diseases.

As one of the major active components of *L. japonica*, its polysaccharides have been found to have multiple bioactivities, including antioxidative [9], anticoagulant [10], antiviral [11], immunomodulating [12] and alleviating metabolic syndrome [13]. However, the effects of the polysaccharides derived from *L. japonica* (LJP) on obesity-related fatty liver and the underlying mechanism remain largely unknown. Therefore, in this paper, we investigated the biological effects of LJP on fatty liver, lipid regulators and GM in a high-fat diet (HFD)-fed mouse model in the hope of illuminating their relationships.

## 2. Results

### 2.1. Characterization of LJP

**Figure 1:**
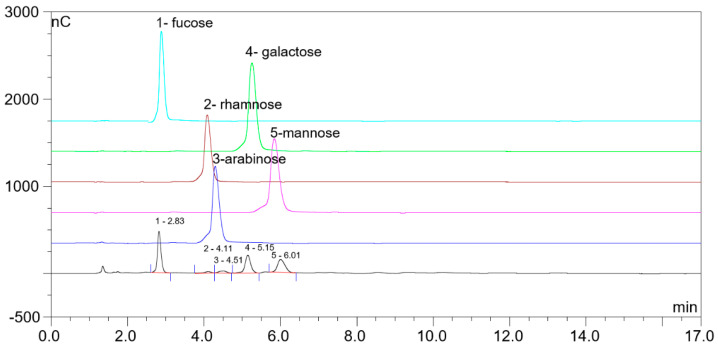
The monosaccharide composition of polysaccharides from *L. japonica* (LJP) detected by HPLC. The black line represents the chromatogram for LJP, and colored lines represent standards for corresponding monosaccharides. 1, fucose; 2, rhamnose; 3, arabinose; 4, galactose; 5, mannose.

LJP was extracted with water. The monosaccharide composition of LJP was determined by HPLC. As shown in Figure 1, LJP was composed of fucose, rhamnose, arabinose, galactose and mannose, the proportion of which was 40.6%, 1.4%, 2.0%, 27.3% and 26.7%, respectively.

**Figure 2:**
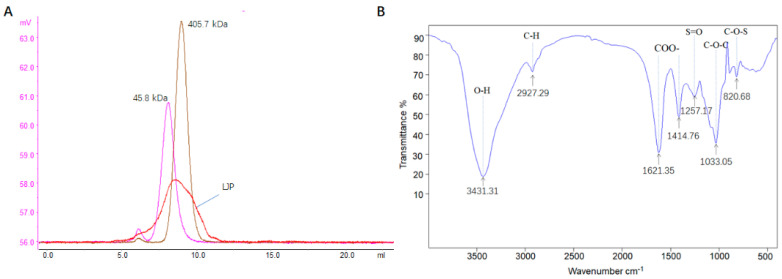
Physicochemical properties of polysaccharide of *L. japonica* (LJP). (**A**) Molecular weight determination. Pink and brown lines represent the chromatograms for the standards of 45.8 kDa and 405.7 kDa, respectively; the red line represents the chromatogram for LJP. (**B**) Fourier transform infrared spectra.

The molecular weight of LJP was about 200 kDa (Figure 2A). The polysaccharide contained carbohydrates (31.30%), ash (19.65%), proteins (4.51%), sulfate (13.70%) and moisture (9.53%). In a general view of the Fourier transform infrared (FT-IR) spectrum of LJP (Figure 2B), the peaks at 3431 cm^−1^ and 2927 cm^−1^ represented the stretching vibration of the O-H in the constituent sugar unit and C–H in the sugar ring, respectively [14]. In addition, polysaccharides presented intense absorption bands at 1621 cm^−1^ and 1414 cm^−1^, which were due to the asymmetrical (C=O) and symmetrical (C-O) stretching vibration of the carboxylate group [15]. The band at ~1257 cm^−1^ (S=O stretching) confirmed the presence of sulfate in LJP, and the absorption band at 820 cm^−1^ suggested a C–O–S stretching vibration of sulfate groups on galactoyranose residues [9].

### 2.2. LJP Improved the Obesity-Related Features in HFD-Fed Mice

**Figure 3:**
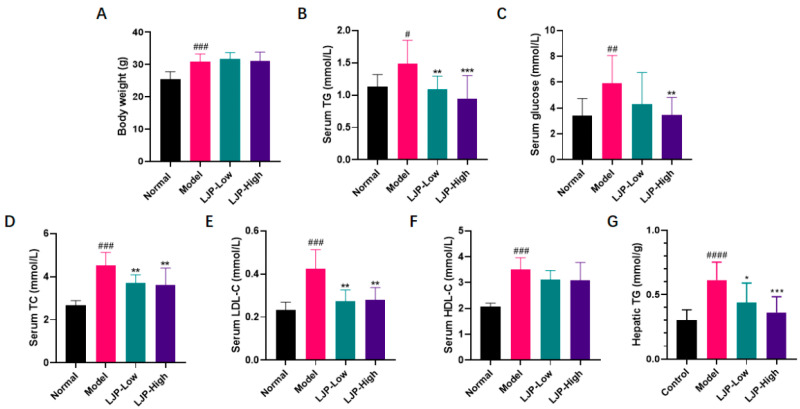
Polysaccharide of *L. japonica* (LJP) improved obese features of high-fat diet-fed mice. (**A**) Body weight. Serum (**B**) triacylglycerol (TG), (**C**) glucose, (**D**) total cholesterol (TC), (**E**) low-density lipoprotein cholesterol (LDL-C), (**F**) high-density lipoprotein cholesterol (HDL-C) and (**G**) hepatic TG. Data are shown as mean ± SD (*n* = 10); # *p* < 0.05, ## *p* < 0.01, ### *p* < 0.001, #### *p* < 0.0001 Model vs. Normal; * *p* < 0.05, ** *p* < 0.01, *** *p* < 0.001 vs. Model.

HFD caused prominent body weight gain and remarkable elevation of serum levels of triacylglycerol (TG), glucose, total cholesterol (TC), high-density lipoprotein cholesterol (HDL-C) and low-density lipoprotein cholesterol (LDL-C) (Figure 3), which largely imitated human obesity. Although LJP did not ameliorate body weight gain or change serum HDL-C level (Figure 3A,F), it significantly reduced serum TG, glucose, TC and LDL-C levels (Figure 3B−E). These results demonstrated that LJP attenuated HFD-fed obesity-related features. Notably, the HFD-elevated hepatic TG was dose-dependently reduced by LJP (Figure 3G), which indicated the protective effect against obesity-related fatty liver.

### 2.3. LJP Reduced Fat Accumulation in the Liver of HFD-Fed Mice

**Figure 4:**
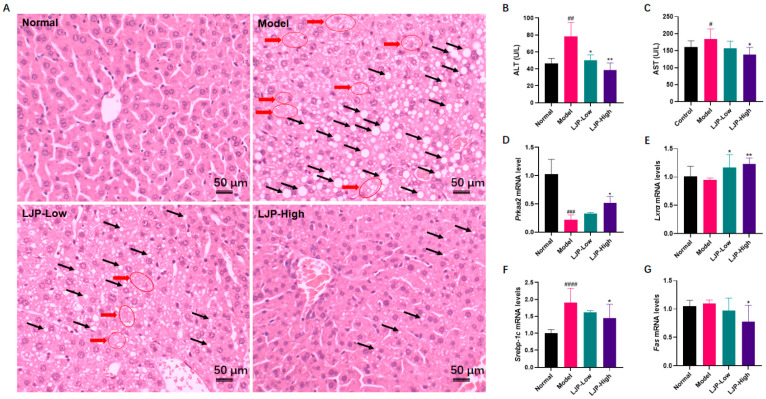
Polysaccharides of *L. japonica* (LJP) improved liver injury of high-fat diet-fed mice. (**A**) Liver sections was stained with haematoxylin and eosin. Black arrows indicate fat deposition in the forms of macrovesicular or microvesicular steatosis; red arrows indicate hepatocellular ballooning. (**B**) Alanine aminotransferase (ALT) and (**C**) aspartate aminotransferase (AST) in the serum. Hepatic mRNA levels of (**D**) *Prkaa2*, (**E**) *Lxrα*, (**F**) *Srebp-1c* and (**G**) *Fas*. Data are shown as mean ± SD (*n* = 10); # *p* < 0.05, ## *p* < 0.01, ### *p* < 0.001, #### *p* < 0.0001 Model vs. Normal; * *p* < 0.05, ** *p* < 0.01 vs. Model.

To assess the impact of LJP on hepatic pathological changes caused by HFD, liver sections were stained with haematoxylin and eosin (H&E) and the results showed that macrovesicular steatosis and ballooning degeneration were severe in the liver of HFD-fed mice but were reduced dose-dependently in the liver of mice treated with LJP. In the LJP-High group, steatosis was largely ameliorated, and fat deposition was mostly microvesicular. Furthermore, hepatocellular ballooning was not observed in the LJP-High group (Figure 4A). Additional determination of serum enzymes verified the role of LJP in protection from liver injury. As shown in Figure 4B,C, compared with those in the Normal group, the activities of serum alanine aminotransferase (ALT) and aspartate aminotransferase (AST) were significantly higher in HFD-fed mice. They were reduced in the LJP-Low and LJP-High groups. In addition, dose-dependent differences were observed between these two groups.

To explore the mechanisms involved in the LJP-mediated improvement of disrupted hepatic lipid metabolism in HFD-fed mice, changes in the mRNA levels of lipogenic regulating genes (protein kinase AMP-activated catalytic subunit α2 (*Prkaa2*), liver X receptor α (*Lxrα*), lipid metabolism-regulating (sterol regulatory element-binding protein 1c (*Srebp-1c*) and fatty acid synthase (*Fas*)) were analyzed. As a result, hepatic mRNA levels of *Prkaa2* were dramatically decreased by HFD and restored by LJP in a dose-dependent manner (Figure 4D). *Lxrα* expression was not altered by HFD, but significantly elevated by LJP, especially in the high-dose group (Figure 4E). LJP treatment significantly decreased the HFD-elevated mRNA level of *Srebp-1c* (Figure 4F). The mRNA level of *Fas* was significantly decreased by high-dose LJP treatment (Figure 4G).

### 2.4. LJP Modulated Gene Expression of Cholesterol Metabolic Genes

It was observed that HFD elevated serum cholesterol levels (TC, HDL-C and LDL-C), and LJP showed a significant modulating effect, especially on TC and LDL-C. Therefore, to explore the mechanism underlying LJP’s modulating cholesterol metabolism, the mRNA levels of the cholesterol regulators, including 3-hydroxy-3-methylglutaryl–coenzyme A reductase (*Hmgcr*), cholesterol 7α-hydroxylase (*Cyp7a1*) and sterol O-acyltransferase 1 (*Soat1*), as well as the hepatic lipoprotein receptors and related proteins, including low-density lipoprotein receptor (*Ldlr*), LDLR-related protein (*Lrp1*) and scavenger receptor class B type 1 (*Scarb1*), were investigated.

**Figure 5:**
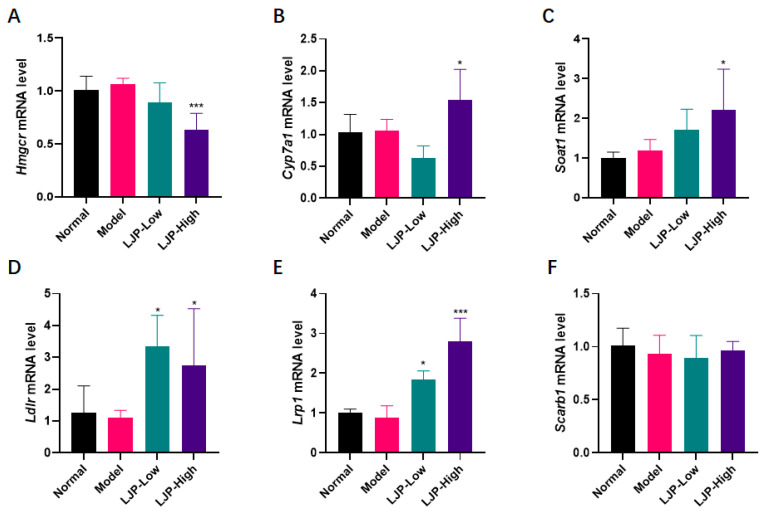
The effect of polysaccharides of *L. japonica* (LJP) on levels of cholesterol-regulating genes. Hepatic mRNA levels of (**A**) *Hmgcr*, (**B**) *Cyp7a1*, (**C**) *Soat1*, (**D**) *Lrp1*, (**E**) *Ldlr* and (**F**) *Scarb1*. Data are shown as mean ± SD (*n* = 10); * *p* < 0.05, *** *p* < 0.001 vs. Model.

As shown in Figure 5, *Hmgcr* was dramatically downregulated by high-dose LJP treatment (Figure 5A). *Cyp7a1* and *Soat1* were upregulated by high-dose LJP treatment (Figure 5B,C). The mRNA level of *Ldlr* was increased by low- and high- dose LJP. *Lrp1* was upregulated by LJP dose-dependently. *Scarb1* expression was not altered.

### 2.5. LJP Changed the Profiles of GM in HFD-Fed Mice

GM has been considered a major environmental factor that plays an important role in the development of obesity and liver damage. In this study, we evaluated the effects of LJP on GM by the multiplex sequencing of 16S rRNA in HFD-fed mice. 

**Figure 6:**
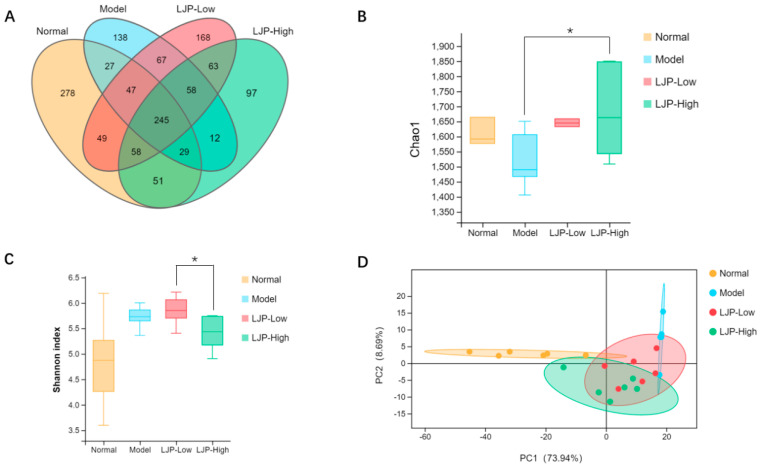
Polysaccharides of *L. japonica* (LJP) altered gut microbiota (GM) composition in high-fat diet-fed mice. (**A**) Venn diagram showing the distribution of operational taxonomic units (OTUs) among different groups. (**B**) Chao1 richness index of GM. (**C**) Shannon diversity index of GM. (**D**) PCA plot of microbial communities based on the OUT level. Data are shown as mean ± SD (*n* = 5 for Model and *n* = 6 for the other groups); * *p* < 0.05.

The Venn diagram showed that all of the groups had unique and shared operational taxonomic units (OTUs) (Figure 6A). Meanwhile, the values of Chao1 richness tended to decrease with HFD (*p =* 0.25) and were significantly increased by LJP treatment (Figure 6B). However, HFD seemed to upregulate the Shannon diversity index, while LJP tended to decrease the value (Figure 6C). Additionally, the principal component analysis (PCA) ordination plot showed that the clusters of GM in the Normal group were clearly separated from those in the HFD group, whereas the clusters of GM in the LJP groups were between those in the Normal group and the HFD group (Figure 6D). Collectively, these results imply that LJP treatment modulates the GM of HFD-fed mice. 

### 2.6. Composition of GM Modulated by LJP in HFD-Fed Mice

**Figure 7:**
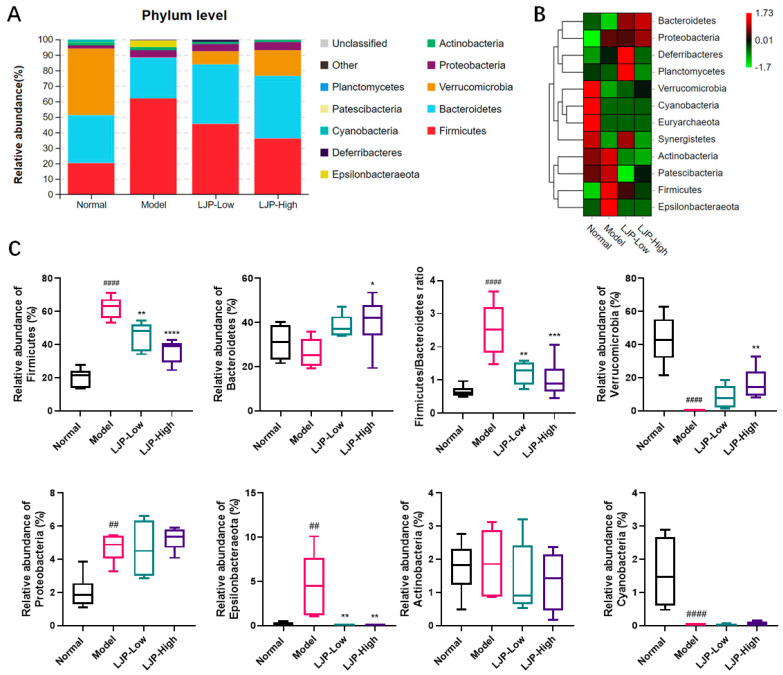
Polysaccharides of *L. japonica* (LJP) modulated gut microbiota (GM) composition at the phylum level in high-fat diet-fed mice. (**A**) Bacterial taxa relative abundance of all the groups at the phylum level. (**B**) Heat map of cluster stacking at the phylum level. (**C**) The relative abundances of Firmicutes, Bacteroidetes, and the ratio of Firmicutes to Bacteroidetes, Verrucomicrobia, Proteobacteria, Epsllonbacteraeota, Actinobacteria and Cyanobacteria. Data are expressed as mean ± SD (*n* = 5 for Model and *n* = 6 for the other groups). ## *p* < 0.01, #### *p* < 0.0001 Model vs. Normal; * *p* < 0.05, ** *p* < 0.01, *** *p* < 0.001, **** *p* < 0.0001 vs. Model.

We analyzed the phylotypes of GM among different groups at the phylum and genus levels. At the phylum level, the results showed that GM were mainly composed of Firmicutes, Bacteroidetes and Verrucomicrobia (Figure 7A). As shown in Figure 7A,B, the proportion of Verrucomicrobia was significantly decreased in the Model group, whereas the proportion was dose-dependently increased by LJP treatment. HFD administration significantly elevated the proportions of Firmicutes and Epsllonbacteraeota, while LJP treatment significantly reduced the proportions of these two phyla in HFD-fed mice. Meanwhile, the Firmicutes to Bacteroidetes (F/B) ratio was reduced from 2.52 ± 0.80 (Model) to 1.02 ± 0.56 (LJP-High) (Figure 7C). Since a lower F/B ratio was observed in lean individuals when compared to their obese counterparts in mice and humans [16], our data indicated the beneficial effect of LJP with respect to obesity. 

**Figure 8:**
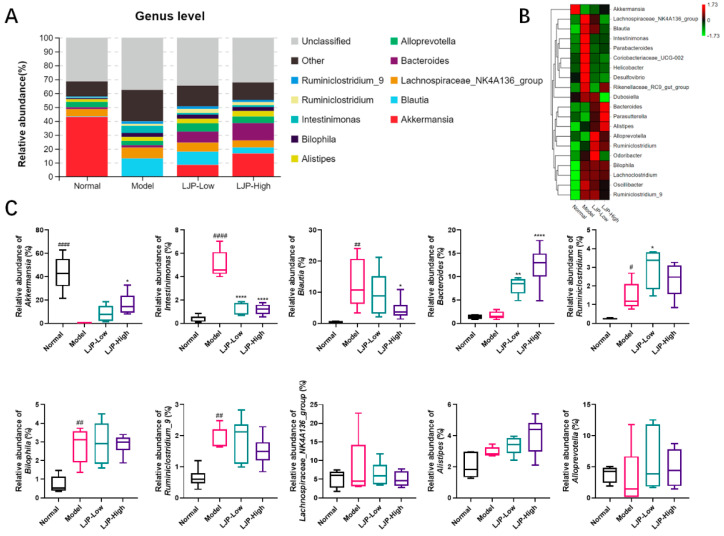
Polysaccharides of *L. japonica* (LJP) modulated gut microbiota (GM) composition at the genus level in high-fat diet-fed mice. (**A**) Bacterial taxa relative abundance of all the groups at the genus level. (**B**) Heat map of cluster stacking at the genus level. (**C**) The relative abundances of *Akkermansia*, *Intestinimonas*, *Blautia*, *Bacteroides*, *Ruminiclostridium*, *Bilophila*, *Ruminiclostridum_9*, *Lachnospiraceae_NK4A136_group*, *Alistipes* and *Alloprevotella*. Data are expressed as mean ± SD (*n* = 5 for Model and *n* = 6 for the other groups). # *p* < 0.05, ## *p* < 0.01, #### *p* < 0.0001 Model vs. Normal; * *p* < 0.05, ** *p* < 0.01, **** *p* < 0.0001 vs. Model.

At the genus level, compared to the Normal group, the Model group had higher abundances of *Intestinimonas*, *Blautia*, *Ruminiclostridium*, *Bilophila* and *Ruminiclostridum_9*, but a much lower abundance of *Akkermansia* (Figure 8A). From the heat map of cluster stacking, it was observed that LJP treatment switched the abundances of the following bacteria to levels similar to those in the Normal group: *Lachnospiraceae_NK4A136_group*, *Blautia*, *Intestinimonas*, *Parabacteroides*, *Corobacteriaceae_UVG-002*, *Helicobacter*, *Desulfovibrio* and *Odoribacter* (Figure 8B). The histogram also shown that the relative abundances of these bacteria were in accordance with the above results (Figure 8C). Thus, LJP treatment partly counteracted the influence of HFD on the abundance profile of the above genera. It should be noted that the abundance of *Bacteroides* was not altered by HFD but was elevated by LJP in a dose-dependent manner (Figure 8A,C). Taken together, these results suggest that LJP regulates bacterial composition in HFD-fed mice, which might be beneficial in ameliorating HFD-induced obesity and liver damage. 

### 2.7. LJP Modulated Gut Fermentation Products

**Figure 9:**
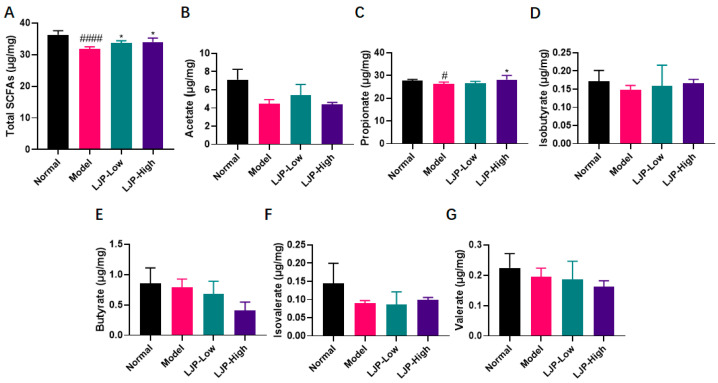
Polysaccharides of *L. japonica* (LJP) changed the production profile of short-chain fatty acids (SCFAs) in HFD-fed mice. Concentrations of (**A**) total SCFAs, (**B**) acetate, (**C**) propionate, (**D**) isobutyrate, (**E**) butyrate, (**F**) isovalerate, and (**G**) valerate were determined. Data are shown as mean ± SD (*n* = 5 for Model and *n* = 6 for the other groups); # *p* < 0.05, #### *p* < 0.0001 Model vs. Normal; * *p* < 0.05 vs. Model.

Short-chain fatty acids (SCFAs) are the main metabolic products of anaerobic bacterial fermentation in the intestine [17]. To investigate the influence of LJP on the metabolic activity of GM, the concentrations of six SCFAs (acetate, propionate, isobutyrate, butyrate, isovalerate and valerate) in cecal contents were detected with GC–MS, and the results were shown in Figure 9. In this trial, acetate, propionate and butyrate were found to predominate in all diet groups. Furthermore, total concentration of SCFAs was significantly reduced by HFD and recovered with LJP treatment. In detail, HFD lowered the concentration of propionate significantly. Administration of 5% LJP significantly restored the propionate concentration in HFD-fed mice. The content of the other five SCFAs showed no statistical differences. The inclusion of LJP in the HFD diet rebalanced these compounds.

## 3. Discussion

NAFLD is now the most common chronic liver disease, and its prevalence is rapidly increasing worldwide. Hepatocellular steatosis is the hallmark of NAFLD [18]. Steatosis, lobular inflammation, and hepatocellular ballooning are all necessary for the diagnosis of nonalcoholic steatohepatitis (NASH), the severe form of NAFLD. In this report, no typical lobular inflammation was observed in all groups. However, hepatocellular steatosis and ballooning (Figure 4A) were observed in the liver samples of HFD-fed mice, but they were both attenuated by LJP treatment in a dose-dependent manner, the mechanism underlying which might be the modulation of the fat mass regulators, including AMPK, LXRα, SREBP-1c and FAS, evidenced by the alteration of LJP on corresponding genes (Figure 4D–G). Dyslipidemia is a risk factor for both cardiovascular disease and NAFLD [19]. It is notable that the gene expression of key regulators of cholesterol homeostasis was altered by LJP treatment. *Hmgcr*, which encodes the principal rate-limiting enzyme in cholesterol biosynthesis HMGCR, was aggressively reduced by the inclusion of 5% LJP in the diet (Figure 5A). *Cyp7a1*, which encodes the rate-limiting enzyme of bile acid synthesis (Figure 5B), was also elevated by high-dose LJP, which might promote the conversion of cholesterol to bile acids. Hepatic cells acquire exogenous cholesterol from lipoproteins [20]. LDL, which contains a large amount of cholesterol in an esterified form, is first bound at the cell surface by LDLR, and internalized by endocytosis, which reduces serum LDL levels [21]. Similarly, LRP1 and SCARB1 are responsible for the binding and endocytosis of IDL [22] and HDL [23], respectively. In this report, LJP upregulated *Ldlr* and *Lrp1* expression levels (Figure 5D,E) and downregulated serum TC and LDL-C levels in HFD-fed mice (Figure 3D,E). The *Scarb1* was not altered by LJP; serum HDL-C levels were not changed in HFD-fed mice either (Figure 5F). The modulation by LJP of hepatic gene expression of the lipoprotein receptors matched the results of the determination of serum cholesterol levels. Interestingly, *Soat1*, which encodes an enzyme that catalyzes the formation of fatty acid-cholesterol esters from cholesterol and acyl-CoA molecules, was upregulated by LJP (Figure 5C). The upregulation of *Soat1* could be explained by the adaptation of hepatic cells to the elevation in cholesterol availability. Additionally, LXRα was reported to promote bile acid synthesis from cholesterol by positively regulating the transcription of *Cyp7a1* [24]. In this report, LJP upregulated *Lxrα* expression dose-dependently (Figure 4E), which might promote bile acid synthesis, leading to lowered serum cholesterol levels. Taken together, these findings showed that LJP modulated hepatic gene expressions related to cholesterol metabolism and that it might promote serum cholesterol transportation into the liver and accelerate the conversion of cholesterol into bile acid.

Recent studies have demonstrated that HFD is correlated with the imbalance of GM, which exacerbates the progression of HFD-induced obesity [25,26]. In the present study, there were distinct OTUs among the different groups (Figure 6A). LJP upregulated the species richness characterized by the Chao1 index (Figure 6B) but downregulated the diversity characterized by the Shannon index in HFD-fed mice (Figure 6C), which indicated that LJP treatment increased the species number in the GM, but not evenly. The PCA plot revealed that GM characteristics in LJP groups were between those in the HFD-fed group and those in the Normal group (Figure 6D). These findings show that GM composition in HFD-fed mice was altered by LJP treatment. LJP can be fermented by GM as substrates, which can in turn alter GM composition. Additionally, *L. japonica* shows laxative properties [27], which could further contribute to GM regulation. 

It this report, Firmicutes, Bacteroidetes and Verrucomicrobia were the most abundant phyla in the intestinal microbiota of the Normal group. LJP elevated the abundance of Bacteroidetes and reduced the abundance of Firmicutes, and thus reduced the F/B ratio (Figure 7A–C). The F/B ratio was used as a dysbiosis parameter in disease [16]. Therefore, LJP modulated GM dysbiosis caused by HFD. Moreover, Verrucomicrobia was dramatically reduced by HFD and recovered by LJP in a dose-dependent manner. Particularly, Epsilonbacteraeota seemed to be characteristic of the HFD group in this study, and the elevation of its abundance was fully abolished by LJP treatment, even in the low-dose group (Figure 7C). At the genus level, it is found that LJP increased *Akkermansia* and *Bacteroides* abundances and decreased *Intestinimonas* and *Blautia* abundances (Figure 8A–C). It has been demonstrated that *Akkermansia* colonizes the gut mucus layer, which degrades mucin and maintains intestinal barrier integrity. The genus *Akkermansia* falls under the Verrucomicrobia phylum [28]. In the present study, the *Akkermansia* abundance alteration by HFD and LJP coincided with that of the Verrucomicrobia (Figure 7C and Figure 8C), which indicated *Akkermansia* as the representative genus of the Verrucomicrobia phylum.

The pharmacokinetics of fucoidans from *L. japonica* have been reported in rabbits, rats and mice via different routes of administration [29]. With peroral administration in rats, when the dose of fucoidans (molecular weight: 100 kDa) from *L. japonica* was at 20 mg/kg, C_max_ was 7.33 μg/mL, T_max_ was 2 h and AUC was 42.69 g·h/mL [30]. The bioavailability of fucoidan was about 8.91% [30], which suggests that a small portion of the polysaccharide could be absorbed into circulation. Studies also showed that the polysaccharide was distributed in the liver, lungs, spleen and kidneys [29], which implies that the polysaccharide distributed in the liver might contribute to hepatic mRNA alteration. The LJP investigated in this paper has a molecular weight of 200 kDa, which should be absorbed with greater difficulty by the intestinal tract. In other words, most of the LJP might undergo microbial fermentation. SCFAs, catabolic end products from intestinal microbial fermentation, are closely related to the occurrence and development of obesity and related metabolic diseases [31]. Additionally, the content of SCFAs reflects the structure of the intestinal flora [32]. In this report, compared with the HFD group, LJP treatment enriched the *Bacteroides* and *Akkermansia* genera (Figure 8C), which have been reported to produce propionate [33,34]. Notably, several studies pointed to *Bacteroides* as the main propionate producer [35,36]. Furthermore, it was reported that propionate is metabolized in the liver and decreases hepatic lipogenesis, reduces serum cholesterol and is potent in triggering enteroendocrine L-cells to signal a satiety response [37]. In addition, studies showed that propionate lowered cholesterol synthesis rates by decreasing HMGCR [38,39]. Importantly, AMPK has been found to be activated by propionate [40,41,42]. Furthermore, AMPK has also been reported to inhibit HMGCR and reduce cholesterol levels in hepatocytes [43]. It is thus inferred that the cholesterol-lowering effect of LJP was mediated through the AMPK pathway by propionate, since *Prkaa2*, which encodes the AMPK subunit α2, was significantly upregulated by LJP treatment in this study (Figure 4D). Therefore, LJP’s effect on the reduction of plasma concentrations of cholesterol could be attributed to the elevation of propionate producing bacteria like *Bacteroides* and *Akkermansia*. Collectively, LJP treatment could modulate HFD-induced GM dysbiosis and alter the fermentation products to ameliorate HFD-induced dyslipidemia.

Recent papers have described similar positive effects of *L. japonica* on HFD-induced metabolic disorders via regulating GM. Li et al. reported that purified *L. japonica* polysaccharide ameliorated HFD-induced insulin resistance and associated metabolic disorders via regulating GM, and the claim was supported by fecal transplantation [44]. The above-mentioned study focused on HFD-induced systemic inflammation and insulin resistance. As in our study, the potential role of *Akkermansia* was highlighted. 

Another study found that the fine powder of *L. japonica* has a protective effect against lipid metabolism disorders in HFD-fed rats. Liver metabolomics was analyzed, and the metabolic pathway enrichment analysis of hepatic metabolites indicated that primary bile acid biosynthesis and cysteine and methionine metabolism were the two main metabolic pathways altered by *L. japonica* consumption [45]. As in our study, the hepatic mRNA alteration of *Srebp-1c*, *Hmgcr* and *Cyp7a1* was found. The difference was that polysaccharides derived from *L. japonica* rather than fine power were used in our study. 

Zheng et al. prepared low-molecular alginate from *L. japonica* (110 kDa) and investigated the beneficial effect on HFD-fed mice. The role of GM alteration was confirmed by fecal transportation from alginate-fed mice [46]. The authors emphasized its advantages of lower molecular weight and better solubility. BALB/c mice were used, and the alginate was added into drinking water. In our study, C57BL/6 mice were used, and the polysaccharide (200 kDa) was added into HFD. The two studies had similar results, like the alteration of *Bacteroides*, but the regulation of *Akkermansia* was not mentioned by Zheng et al., which may be due to the differences of GM composition between BALB/c and C57BL/6 mice.

## 4. Materials and Methods

### 4.1. Preparation of LJP

*L. japonica*, harvested at Dongshan, Fujian, China in April 2019, was supplied by Fujian Yuanyang Algae Industry Co., Ltd. The material was cut into strips and washed 4 times to remove the salt, followed by two rounds of delipidation (25 °C, 4 h, seaweed: 95% ethanol 1:20 (*w:v*)). The material was then dried and powdered. About 1 kg of the material powder was extracted by water for two cycles (100 °C, 3 h, seaweed:water 1:60 (*w:v*)). The extract was collected, concentrated to about 10 L and spray-dried, then LJP was prepared. The yield of LJP was about 9.8%. Dextran analytical standards with a molecular mass of 45.8 and 405.7 kDa were purchased from the American Polymer Standards Corporation (Mentor, OH, USA).

### 4.2. Characterization of LJP

To determine the monosaccharide composition of LJP, the polysaccharides were hydrolyzed into monosaccharides by dissolving 1 mg LJP in 2 mL hydrochloride solution, followed by incubation at 100 °C for 3 h. The hydrolysis solution was then diluted to 10 mL and determined with an ICS-3000 ion chromatograph (Dionex, Sunnyvale, CA, USA) equipped with a Dionex CarboPac PA10 column (250 mm × 4 mm, Thermo Fisher Scientific, Waltham, MA, USA). Sodium hydroxide aqueous solution and water (10:90, 1 mL/min) was used as the mobile phase. Then, the monosaccharides, such as fucose, rhamnose, arabinose, galactose and mannose, were detected and confirmed by the monosaccharide standards [47].

The total sugar content was determined according to the phenol–sulfuric acid method, using glucose as the standard [48]. The moisture, protein and ash contents of LJP were determined using AOAC standard methods [49]. The content of sulfate group was measured using the barium sulfate turbidimetric method [50]. The molecular weight of LJP was determined using an AKTA purifier 10 (GE Healthcare, Uppsala, Sweden) equipped with a TSKgel GMPWXL column (300 mm × 7.8 mm, Tosoh Bioscience, Torino, Italy), with water as the mobile phase (1 mL/min). The polysaccharide sample was ground in a mortar, blended with dried KBr and pressed into pellets, followed by FT-IR analysis at 400 cm^−1^ to 4000 cm^−1^ using an FT-IR spectrometer (Tansor27, BRUKER, Karlsruhe, Germany).

### 4.3. Animal Study

Male 5-week-old C57BL/6 mice were purchased from the Animal Centre of Xiamen University (Fujian, China). The mice were housed and maintained in a temperature- and humidity-controlled environment with 12 h light/dark cycle.

The mice (19.8–22.6 g) were randomly divided into four groups (10 mice/group) individually. Group 1 was the normal control group, in which the mice were fed with a control diet (Normal, 10% kcal from fat, D12450J, Research Diets, New Brunswick, NJ, USA). Group 2 was the model group, in which the mice were fed with an HFD (Model, 60% kcal from fat, D12492, Research Diets). Group 3 was a treatment group, in which the mice were fed with an HFD with 2.5% (*w*/*w*) LJP (LJP-Low). Group 4 was another treatment group in which the mice were fed with an HFD with 5% (*w*/*w*) LJP (LJP-High). After 8 weeks of treatment, the mice were anaesthetized, and blood was collected via the retro orbital sinus. Then, the mice were sacrificed and a small portion of the liver was removed and rapidly frozen with liquid nitrogen for hepatic RNA isolation. For each group, cecal samples were collected randomly (*n* = 5 for Model, and *n* = 6 for the other groups) and stored in liquid nitrogen before further analysis. Efforts were made to minimize animal suffering as much as possible. The experimental protocol was approved by the institutional animal care and use committee of the Third Institute of Oceanography, Ministry of Natural Resources (ethical committee approval number: TIO-IACUC-10-2019-10-23), and all animals received humane care according to the National Institutes of Health (USA) guidelines.

### 4.4. Quantitative RT-PCR Analysis

Total RNA was extracted from the liver samples using Trizol reagent (Biouniquer Technology Co., Ltd., Nanjing, China), according to the manufacturer’s protocol. An equal amount of RNA (500 ng) from each sample was then converted to cDNA using PrimeScript™ RT Reagent Kit (RR037A, Takara, Shiga, Japan), according to the manufacturer’s instructions. Quantification of gene expression was performed using Platinum™ SYBR™ Green qPCR SuperMix-UDG (11744-500, Invitrogen, Carlsbad, CA, USA), according to the manufacturer’s instructions. The relative mRNA expression level was measured by the 2^−ΔΔCt^ method and normalized to *Gapdh*. Sequences of the primers used in real-time qPCR are shown in Table 1.

### 4.5. Histological Evaluation

Fresh liver samples were fixed in paraformaldehyde, embedded in paraffin and then sectioned at 5 μm. The sections were stained with HE, and digital images were obtained using a Nikon E80i microscope (Nikon, Tokyo, Japan).

### 4.6. Biochemical Analysis

The levels of AST, ALT, TG, TC, LDL-C, HDL-C and glucose in the serum were detected with the Mindray BS-240VET Chemistry Analyzer (Mindray, Shenzhen, China). The hepatic TG was measured using a commercial kit (A110-2, Nanjing Jiancheng, Nanjing, China).

### 4.7. Analysis of Intestinal Microbiota

The bacterial DNA of each cecal sample was extracted using a HiPure Stool DNA Kit B (Magen, Shanghai, China), following the manufacturer’s instructions. The DNA extractions were quantified by ultraviolet spectroscopy. The 16S rDNA V3–V4 region were amplified by PCR (94 °C for 2 min, followed by 30 cycles at 98 °C for 10 s, 62 °C for 30 s, and 68 °C for 30 s and a final extension at 68 °C for 5 min) using primers 341F: CCTACGGGNGGCWGCAG; 806R: GGACTACHVGGGTATCTAAT. Amplicons were extracted from 2% agarose gels and purified using the AxyPrep DNA Gel Extraction Kit (Axygen Biosciences, Union City, CA, USA), according to the manufacturer’s instructions, and quantified using the ABI StepOnePlus Real-Time PCR System (Life Technologies, Foster City, CA, USA). Purified amplicons were pooled in equimolar and paired-end sequenced (PE250) on an Illumina platform according to the standard protocols. Raw reads were further filtered using FASTP (version 0.18.0) [51]. Paired end clean reads were merged as raw tags using FLASH (version 1.2.11) [52], with a minimum overlap of 10 bp and mismatch error rates of 2%. Then, the raw tags were quality filtered and chimeric sequences removed to acquire the effective tags, which were clustered into OTUs with ≥97% identity cutoff using UPARSE software (version 9.2.64) [53]. PCA was performed in the R project Vegan package (version 2.5.3) [54]. The Chao1 richness and Shannon diversity index were conducted with QIIME software (version 1.9.1, University of Colorado, Denver, CO, USA). The dominant bacteria were analyzed mainly at the phylum and genus levels using the R project. The heat map of cluster stacking was calculated using the R package and generated using Omicsmart (Genedenovo Biotechnology Co. Ltd., Guangzhou, China), a dynamic real-time interactive platform for data analysis.

### 4.8. Quantification of SCFAs

The supernatant was prepared by homogenizing 0.02 g cecal contents in 1.0 mL sodium hydroxide solution (5 mmol/L) in ice water, followed by centrifugation at 12,000× *g* for 10 min at 4 °C. The supernatant was then subjected to the derivatization procedure. A 500-μL aliquot of propyl chloroformate was carefully added to 300 μL sample. To release the gases generated by the reaction, the tube lid was kept open for 1 min, then the lid was closed, and the mixture was vortexed. Next, 300 μL hexane was added and vortexed, and the tubes were centrifuged at 12,000× *g* for 5 min. The upper hexane phase was transferred into an autosampler vial for analysis. An Agilent 7890/5977A GC-MS (Santa Clara, CA, USA) system equipped with an HP-5 capillary column (30 m × 0.25 mm × 0.25 μm) was supplied for determining the concentrations of SCFAs, including acetate, propionate, butyrate, isobutyrate, valerate and isovalerate in the cecal contents. 

### 4.9. Statistical Analysis

Data are presented as means ± SD. Significance of differences was determined by one-way ANOVA with the post hoc Tukey test. Histograms were created using GraphPad Prism 8 software (San Diego, CA, USA). Bioinformatics analysis, including species taxonomy, richness and diversity analyses, was performed using Omicsmart (Genedenovo Biotechnology Co. Ltd., Guangzhou, China). A *p*-value < 0.05 indicates statistically significant differences.

## 5. Conclusions

In conclusion, this study suggested that LJP attenuates NAFLD by modulating both hepatic gene expression of lipid regulators and GM dysbiosis in HFD-treated mice. LJP improved energy homeostasis by elevating the fecal content of propionate, which should be attributed to the modulation of GM. Our findings provide a new insight into the use of LJP for the prevention of obesity-induced fatty liver through adjusting lipid regulators and GM composition.

## Figures and Tables

**Table 1:** The sequences of RT-PCR gene-specific primers.

Genes	Forward Primer	Reverse Primer
*Prkaa2*	GTGATCAGCACTCCGACAGA	TCTCTGGCTTCAGGTCCCTA
*Lxrα*	ATCGCCTTGCTGAAGACCTCTG	CTGCTTTGGCAAAGTCTTCCCG
*Fas*	CATGACCTCGTGATGAACGTGT	CGGGTGAGGACGTTTACAAAG
*Srebp-1c*	GGAGCCATGGATTGCACATT	GGCCCGGGAAGTCACTGT
*Hmgcr*	GCTCGTCTACAGAAACTCCACG	GCTTCAGCAGTGCTTTCTCCGT
*Cyp7a1*	TCTCCCTTGAGGGTCTCTCC	GTGGCAACCTCCTGCAATTC
*Soat1*	TTCGGCCTTGTGCGACTTAT	AAGTCTAACCCGAGGCAAGC
*Lrp1*	GCGATGAGAGTGTCCGCATA	CGTGTGCCAGTTAGTCCAGT
*Ldlr*	CCAATCGACTCACGGGTTCA	ACAGTGTCGACTTCTCTAGGC
*Scarb1*	TTTGTTGGGATGAACAACTC	GTCCCATTGATCATGTTACAC
*Gapdh*	GGTGAAGGTCGGTGTGAACG	CTCGCTCCTGGAAGATGGTG

## Data Availability

All data supporting the conclusions of this article are included in this article.
